# AI and digital justice in EU labor law. A comparative study on predictive tools and judicial transformation

**DOI:** 10.3389/frai.2025.1742239

**Published:** 2026-01-29

**Authors:** Marianna Molinari, Marco Giacalone

**Affiliations:** 1 Department of Legal Studies, University of Bologna, Bologna, Italy; 2 Department of Computer Science, University of Turin, Turin, Italy; 3 Department of Private and Economic Law, Vrije Universiteit Brussel, Brussels, Belgium

**Keywords:** artificial intelligence, digital justice, European litigation, labor law, predictive tools

## Abstract

This paper examines how artificial intelligence (AI) and digital tools are reshaping European labor law litigation, particularly in redundancy disputes. Conducted within the I-Tools To Design And Enhance Access To Justice (IDEA) project, it draws on a comparative survey across six Member States—Belgium, Croatia, the Czech Republic, Estonia, Italy, and Lithuania—to identify best practices in digitalized court systems. The findings point to uneven digital maturity: Estonia and Lithuania lead in digital development (8.4/10; 8.0/10) and show more favorable attitudes toward predictive justice (6.0/10; 5.8/10); whereas Belgium, Croatia, the Czech Republic, and Italy, despite having digital tools, struggle to integrate them into routine legal workflows, reinforcing greater resistance to predictive justice. Although digital justice can improve access and efficiency, concerns persist around fairness, judicial trust, and ethical safeguards. Progress, the study suggests, depends on participatory governance, clear regulation, and the careful integration of technology into procedural frameworks. Building on these insights, the current phase of IDEA positions AI as a regulatory instrument that structures access to justice and guides user behavior. The consortium is developing a legal chatbot to guide workers and employers toward the most suitable resolution pathway—negotiation, mediation, or litigation—based on context, cost, and procedural guarantees. The chatbot tailors its responses in accordance with prevailing legal trends and the reasoning behind case law. A pilot in three Member States (MSs) will test its potential to enhance transparency, empower users, and promote proportional, informed dispute resolution within European labor justice. Against this backdrop, the chapter conceptualizes AI—operating through structured information, triage, and explainability—as a regulatory instrument that can steer behavior ex ante and support compliance ex post in redundancy disputes, complementing adjudication without displacing judicial authority.

## 1 Introduction

Labor law disputes, particularly in the context of economic volatility and structural inequalities, present a critical arena for the application of digital innovation. This relevance is especially pronounced given the structural imbalance inherent in employment relationships: workers often represent the weaker party, particularly when faced with redundancy dismissals that abruptly compromise their economic stability. This asymmetry, especially pronounced in cross-border employment contexts, raises fundamental questions of access to justice, procedural fairness, and judicial adaptability.

This paper forms part of an EU-funded initiative—IDEA: *I-Tools To Design And Enhance Access To Justice*^1^—which seeks to explore and promote digital and predictive justice mechanisms in labor law litigation. The project pursues three main objectives: (i) analyze the judicial systems of six MSs (Belgium, Croatia, Czech Republic, Estonia, Italy, and Lithuania) and identify best practices in digitalization; (ii) develop a legal chatbot that can lead workers toward the most appropriate dispute resolution pathway—negotiation, mediation, or traditional proceedings; and (iii) design a pilot version of the tool to be tested in three selected countries. The study begins by assessing the level of digitalization in each national court system, combining empirical-quantitative indicators with qualitative insights from legal professionals. The methodology blends quantitative survey data with qualitative insights from legal practitioners. This multidimensional approach enables us to examine not only technological adoption but also institutional attitudes and user satisfaction. It underscores the importance of a balanced strategy: one that embraces innovation while safeguarding legal guarantees, transparency, and procedural fairness.

The data reveal substantial cross-country differences in digital capacity and openness to predictive tools. Estonia and Lithuania lead in digital development (8.4/10 and 8.0/10) and show comparatively stronger acceptance of predictive justice (6.0/10 and 5.8/10). In Belgium (4.3/10), Croatia (5.5/10), the Czech Republic (5.1/10), and Italy (4.7/10), support is lower: despite the presence of digital solutions, their limited integration into routine legal work is associated with greater caution toward predictive approaches.

The analytical lens adopted here is not one of technological determinism, but instead of algorithmic regulation: AI-enabled tools can embed legal parameters, surface procedural thresholds, and produce counterfactual guidance that nudges parties toward lawful courses of action while preserving human control (Veale and Borgesius, 2021; Surden, 2014). Framed this way, digital justice becomes a regulatory ecology in which information, design, and oversight jointly structure choices available to workers and employers.

A premise running through this article is that national transposition differences are not peripheral for AI-enabled tools in labor justice; they are what makes such tools legally demanding. EU directives set the frame, but the operative thresholds, procedural steps, competent authorities, and consequences of defective compliance are fixed—often decisively—in national law. A chatbot that guides users through redundancy disputes must therefore be jurisdiction-aware: it must ask the questions that actually matter in that legal order, apply the correct triggers and timelines, and explain its guidance in terms that are meaningful for that forum. The comparative results of the IDEA project are used in that spirit: not only to describe uneven digital maturity, but also to show why trustworthy design depends on making transposition differences visible rather than abstracting them away.

Section 2 provides background on the digital transformation of EU judicial systems, a focus on labor law litigation, and on the growing significance of redundancy dismissal cases. Section 3 presents the comparative analysis of the six MSs involved, outlining best practices and challenges in implementing digital justice. Section 4 discusses the concrete outcomes envisioned by the IDEA Project, based on the study's findings. Section 5 concludes with critical reflections, key takeaways, and suggestions for future works.

## 2 Background

### 2.1 Digital transformation across the EU

According to the 2024 Evaluation Report on European judicial systems - published by the European Commission for the Efficiency of Justice (CEPEJ) and based on 2022 data. This report marks the tenth evaluation cycle, assessing the judicial systems of 44 Council of Europe MSs, along with two CEPEJ observer States, Israel and Morocco. It is also the first edition to incorporate post-COVID data, reflecting the pandemic's impact on the administration of justice across Europe, and communication technology (ICT) remains consistent. Almost all MSs have increased both their average ICT budget per inhabitant and the share of ICT spending, within their overall judicial system budgets. This trend underscores the increasing significance of ICT in the judiciary.

The level of ICT deployment varies widely among states, ranging from as low as 0 out of 10, where 0 corresponds to no deployment, to as high as 8 out of 10, with 10 representing the maximum level of deployment. In contrast, the usage index is generally lower, spanning from 0 to a maximum of 6 out of 10. Interestingly, some countries with the highest deployment scores still show relatively lower usage levels, which may indicate recent ICT advancements or challenges in accurately measuring usage at this stage. In both cases, an increase in ICT usage is anticipated in the coming period.

**Figure 1:**
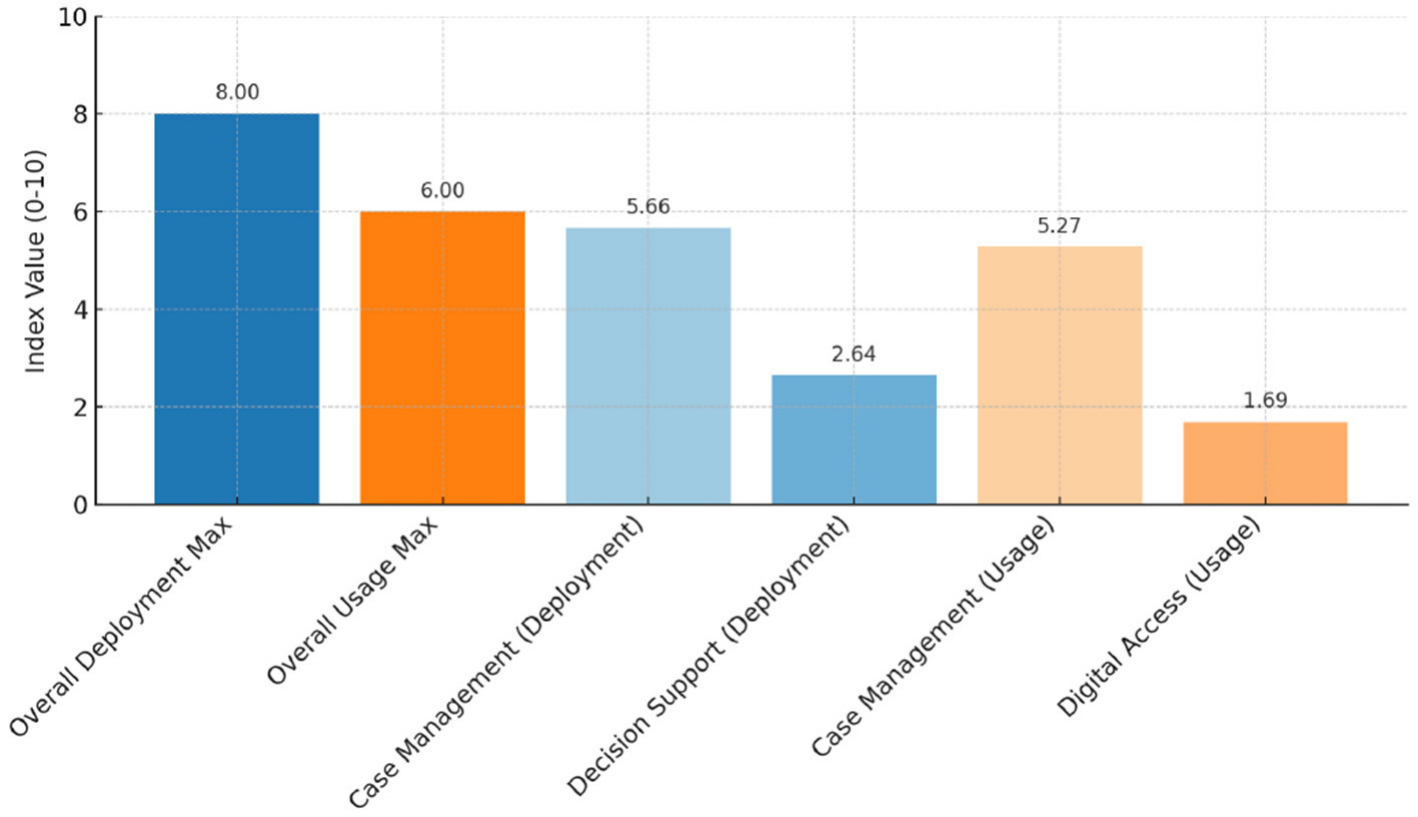
ICT deployment and usage indices in EU judicial systems.

As shown in Figure 1, the European average for the ICT deployment index is highest in the “case management” category (5.66) and lowest in the “decision support” category (2.64), indicating that countries are still primarily focused on establishing fundamental digital infrastructure, such as e-filing and case registration systems. Changes related to a rather basic digital transformation: in other words, the shift from paper to digital. A similar trend is observed in the usage rate, which is highest in the “case management” category (5.27) and lowest in “digital access” (1.69), indicating that the use of e-tools still requires further promotion.

Since the last evaluation cycle and following the COVID-19 pandemic, many MSs and judicial bodies have made significant strides in implementing remote hearings, with 33 states now allowing this option. That cycle also highlights the emergence of innovative tools designed to assist judges, marking the beginning of important developments expected to become more evident in the next evaluation. Early initiatives focus on areas such as automatic anonymization of judgments and specialized translation, where AI is increasingly utilized (Fabri, 2024).

Generally, countries with higher ICT indices tend to have shorter average processing times for cases. Nevertheless, although most MSs conduct some form of evaluation regarding the impact of ICT implementation, assessing these effects remains a complex and possibly premature challenge for the European judiciary (Gascón Inchausti, 2023). This is further reflected in the difficulties some countries face in estimating the deployment or usage rates of certain digital tools.^2^

### 2.2 Labor law litigation across the EU

From 2023 onwards, Europe has experienced several significant labor disputes, including in countries that traditionally have stable industrial relations. The main cause of these conflicts has been the rising costs due to inflation. The sectors most affected include transport, education, health and social care, and manufacturing. Many of these disputes remain unresolved, and conflicts continue in several countries.

**Figure 2:**
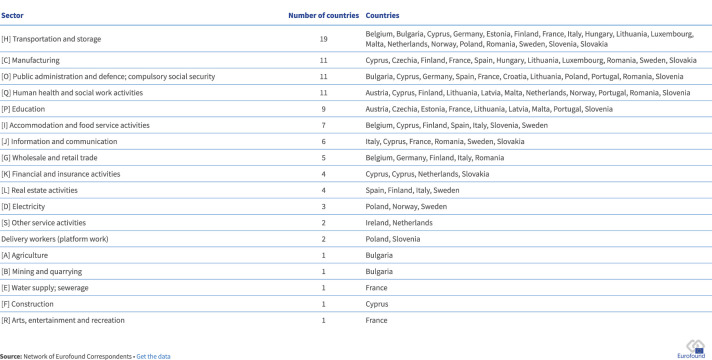
Number of reported significant labor disputes and industrial action by sector, EU Member States and Norway, 2023 (Cojocariu and Sedláková, 2024).

The transport sector experienced the highest number of significant industrial disputes, with conflicts reported in 20 countries (see Figure 2). Notable examples include protests by railway workers in Greece following the Tempi train accident, at Deutsche Bahn in Germany, and at Elron in Estonia. In the aviation industry, employees of Brussels Airlines and Ryanair in Belgium, as well as pilots working for Air Malta and Norsk Luftambulanse in Norway, also engaged in industrial disputes.

The public sector saw widespread industrial disputes in several MSs, with major conflicts among healthcare workers (11 MSs), education staff (9 MSs), and public administration employees—including police, military, and judicial services (11 MSs). These disputes were commonly exacerbated by high inflation and chronic underinvestment in these sectors.

Significant industrial disputes were also reported in the manufacturing sector in eleven countries. Additionally, platform delivery workers launched disputes in Poland against the food delivery platform Pyszne.pl and in Slovenia against food and product delivery platforms Wolt and Glovo.

**Figure 3:**
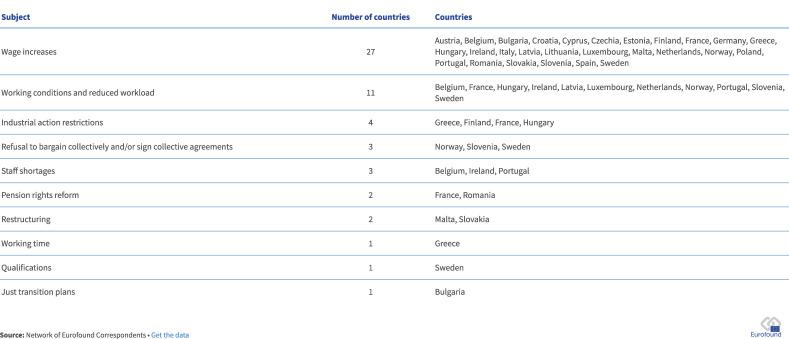
Subjects of industrial action and countries in which they arose, 2023 (Cojocariu and Sedláková, 2024).

The most common cause of significant labor disputes is wages, specifically wages not keeping pace with the rising cost of living due to inflation and higher prices. Wage-related industrial action occurred in all MSs except Denmark, as well as in Norway (see Figure 3). Conflicts often emerged when wage increases failed to match inflation, with some countries, such as Spain, demanding that the costs of inflation be shared equally between employers and workers.

In Italy, a national general strike involving employees from all sectors, including managerial staff, took place in November 2023, primarily in response to the Budget Law. The call for wage increases is aimed at mitigating the effects of inflation, particularly in sectors characterized by high levels of involuntary part-time work, seasonality, and job insecurity, often affecting female workers. In the Netherlands, the staff of the largest trade union, the Federation of Dutch Trade Unions (FNV), went on strike for the first time since its current formation in 2013, demanding automatic price compensation in the new collective labor agreement to address inflation. In Slovakia, workers at UniCredit Bank secured an 8% wage increase following strike action.

Working conditions were the second most common cause of labor law disputes. This category includes demands to reduce class sizes in the education sector (Lithuania and Hungary), opposition to franchising practices that could weaken working conditions in the retail sector (Belgium), cleaning services (Slovenia), protests against increased work pressure due to low staffing in the aviation industry (Belgium), and fire protection services (Ireland). Several protests also focused on pension reforms (France and Romania) and changes to legal regulations, such as the right to strike (Finland).

Important issues triggering disputes include restrictions on industrial action and workers' rights, staff shortages, and workplace changes like restructuring. For example, miners in Bulgaria protested against the government's territorial transition plans submitted to the European Commission. This led to the approval of additional financial support for state-owned mines and a one-year postponement of energy market liberalization to the pastsummer 2025. In Poland and Slovenia, platform workers organized strikes for collective bargaining rights through the newly formed Trade Union of Food Delivery Couriers (Cojocariu and Sedláková, 2024; Colàs-Neila and Yélamos-Bayarri, 2020; Trapmann et al., 2022; Spencer et al., 2022).

### 2.3 Redundancy dismissal: a legal and socioeconomic pressure point

As mentioned above, inflation has contributed to recent labor litigation trends by causing, among other effects, staff shortages and workplace changes, such as restructuring. These disputes typically arise when businesses face economic difficulties and lay off staff, resulting in redundancy dismissals.

Redundancy dismissal occurs when an employee's role is eliminated, not due to their individual actions or performance, but as a result of the company's broader economic, organizational, or production-related needs. This typically arises in cases of restructuring, downsizing, or financial difficulties, where shifts in business strategy or market conditions make certain positions redundant (Lorber, 2017). Unlike dismissals based on misconduct or poor performance, redundancy dismissals are driven by business necessity (International Labour Office, 2000).

In EU law, the triggering of collective consultation duties depends on both numerical and temporal conditions, as well as the notion of establishment. Case law clarifies that thresholds are counted per local unit, rather than across the undertaking as a whole.^3^ These doctrinal subtleties, together with national transposition choices, generate predictable compliance frictions in cross-border settings that are amenable to decision support through rule-aware digital tools (Steiert, 2025).

From a design perspective, Directive 98/59/EC does not operate as a single, “ready-to-compute” rule set. MSs implement it through different threshold formulas and reference periods, different understandings of when consultation is triggered and how its timing must be evidenced, different notification addressees, and different legal consequences where consultation or notification is defective. The practical result is that a restructuring plan that is identical on the facts can generate different legally decisive questions—and different risk profiles—depending on the forum. For an algorithmic-regulatory instrument such as the IDEA chatbot, these divergences are therefore not background comparative context; they are part of the core specification. They determine which facts the system must elicit, which thresholds and timelines it must apply, how it should signal uncertainty, and what kind of explanation is actually useful in that jurisdiction (i.e., grounded in the local doctrinal and procedural logic.

Moreover, at the EU level, taking over all or part of a company does not automatically grant the employer the right to lay off employees. All rights and obligations under employment contracts that exist on the date of the takeover are transferred to the employer.^4^

The employer is also required to continue adhering to the terms of any existing collective agreement until it is terminated, expires, or is superseded by a new agreement. While the duration of this obligation can be shortened in some EU countries, it must last for at least one year.

However, workers' rights are not always fully respected, which often leads to litigation concerning redundancy dismissals. This scenario can become even more complicated in cases of cross-border litigation due to language barriers and disparities between legal systems at the substantive, procedural, and enforcement levels. Considering that there are only a few guiding instruments at the European level, namely: (i) Council Directive 2001/23/EC of 12 March 2001 on the approximation of the laws of the MSs relating to the safeguarding of employees' rights in the event of transfers of undertakings, businesses or parts of undertakings or businesses^5^; and (ii) Council Directive 98/59/EC of 20 July 1998 on the approximation of the laws of the MSs relating to collective redundancies.^6^

## 3 Digital and predictive approaches in labor law litigation: insights from the IDEA project

In light of these complex and evolving labor dynamics — characterized by mounting disputes, structural changes, and socio-economic pressures—it becomes imperative to explore how digital innovation, particularly artificial intelligence and predictive justice, can support and reshape the mechanisms of labor law litigation. Against this backdrop, the next section investigates the perception of AI-driven and general digital tools across EU.

As mentioned above, the investigation is conducted within the framework of the European project *I-Tools To Design And Enhance Access To Justice* (IDEA), carried out across six MSs: Belgium, Croatia, Czech Republic, Estonia, Italy, and Lithuania. The results have been derived through the collaboration of the Consortium, composed as follows: Belgium (Vrije Universiteit Brussel - VUB); Croatia (Sveučilište u Zagrebu – Pravni Fakultet-PFZG); Czech Republic (Masarykova Univerzita-MU); Estonia (Tallinna Tehnikaülikool-TalTech); Italy (Università degli Studi della Tuscia-UNITUS, Università degli Studi di Napoli Federico II-UNINA, Consiglio Nazionale delle Ricerche-CNR); Lithuania (Vilniaus Universitetas-VU); and the Fédération des Barreaux d'Europe-FBE.

### 3.1 The comparative analysis

To advance the current state of research, it is crucial to map the dynamics of digitalization, the adoption of AI, and the emergence of predictive justice in EU labor litigation (Aletras et al., 2016). This includes identifying key challenges, causal relationships, and procedural interdependencies (Medvedeva et al., 2020). Equally important is the evaluation of digitalization measures already implemented in each national context.

To analyse the use of ICT and AI technologies in the courts of the participating countries, an online survey was conducted. The survey began by collecting background information on respondents, including their professional role (e.g., judge, court staff, lawyer, or ICT specialist within a court or Ministry of Justice), country of residence, years of experience, and familiarity with digital technologies.

It then examined which ICT tools are used in labor law proceedings and assessed respondents' satisfaction with these technologies. The survey also investigated the perceived effects of judicial digitalization on labor law in each country and gathered suggestions for improving the use of digital tools in this field. Finally, respondents were asked to share their views on predictive justice, indicating their level of trust or resistance toward such systems.

The results of the survey are presented below, accompanied by heatmaps — representing average values — to aid interpretation.^7^

### 3.2 Country-specific sample sizes and respondent composition

The survey targeted varying numbers of respondents in each country: Belgium (*n* = 105), Croatia (*n* = 89), Czech Republic (*n* = 79), Estonia (*n* = 48), Italy (*n* = 129), and Lithuania (*n* = 87). The composition of respondents differs significantly across countries (percentages rounded to one decimal place). Lawyers constitute the majority in Lithuania (81.6%, *n* = 71), Belgium (67.6%, *n* = 71), and Italy (52.7%, *n* = 68). In contrast, Croatia (47.2%, *n* = 42) and Estonia (22.9%, *n* = 11) display a more balanced professional composition. Regarding judges, Croatia has the highest representation (30.3%, *n* = 27), followed by Italy (27.9%, *n* = 36), Estonia (16.7%, *n* = 8), and Belgium (14.3%, *n* = 15), with Lithuania having the lowest proportion (3.4%, *n* = 3). Court staff are most represented in the Czech Republic (41.8%, *n* = 33) and Estonia (29.2%, *n* = 14), while they remain a minority in the other countries. The “Other” category, which includes professors and researchers, ranges from 12.4% in Croatia (*n* = 11) to 6.3% in the Czech Republic (*n* = 5).

As the survey was conducted by national teams without role-specific quotas, the sample sizes and professional distributions differ by country. Therefore, the comparative analysis and the aggregate figures should be interpreted as indicative, rather than statistically representative. National aggregates are presented as trends and are accompanied by disaggregated data based on professional roles.

To account for this, the results are to be read by professional role and viewed in conjunction with focus groups and interviews for qualitative enrichment, thereby mitigating the potential effects of sample composition.

Moreover, to reduce the heterogeneity across legal professions, the survey instrument normalized the ordinal responses collected on a five-point agreement scale and triangulated them with qualitative materials, an approach consistent with mixed-method standards in courts-and-technology research. ^8^

#### 3.2.1 Belgium

**Figure 4:**
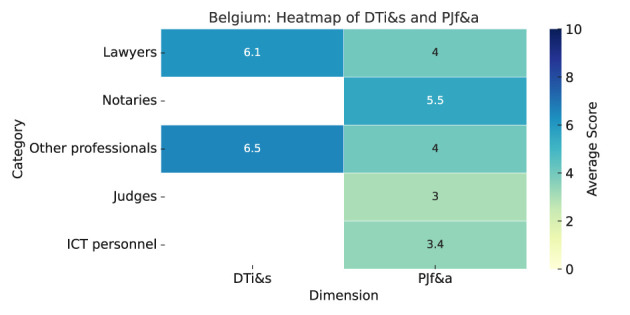
Belgium—Heatmap.

As summarized in Heatmap (Figure 4), the results are as follows:

- **Digital tools impact and satisfaction (DTi&s):** Perceptions of digital tools differ by profession. Lawyers rated their effect on speeding up procedures higher (5.3), than other professionals (4.6). Transparency was also rated slightly better by lawyers (5.1 vs. 4.2). Both groups disagreed with the statement that digital tools had no impact, with scores below 3.5. Overall, lawyers expressed more favorable views across most categories.
  Other professionals reported high satisfaction with automated case allocation and online dispute resolution (mean: 8.0). In contrast, lawyers preferred electronic case management (7.5) and online payments systems (7.2), but were less satisfied with automated allocation (5.4) and online dispute resolution (5.2).
- **Predictive justice familiarity and acceptance (PJf&a):** Familiarity with predictive justice is low among Belgian legal professionals (mean: 2.4). Notaries report the highest familiarity (2.9), followed by other professionals (2.4) and lawyers the lowest (1.8), all below the midpoint of 5. Notaries are most receptive to its use in court decisions (6.1), while lawyers are cautious (4.4), and others are more skeptical (3.7). Notaries show greater optimism across impact areas, seeing the highest benefit in administrative tasks (7.5), followed by lawyers (5.8) and other professionals (5.9). All groups doubt improvements in access to justice. Judges are seen as most resistant to change (nearly 7), especially by other professionals (7.7), while ICT personnel are least resistant (3.1–3.6).

#### 3.2.2 Croatia

**Figure 5:**
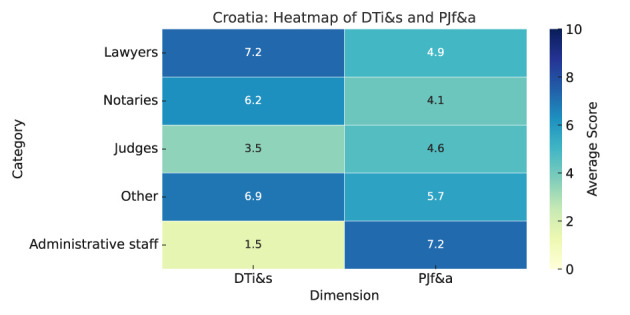
Croatia—Heatmap.

As summarized in Heatmap (Figure 5), the results are as follows:

- **Digital tools impact and satisfaction (DTi&s):** Croatian legal professionals generally view digital tools positively, especially for transparency (notaries 8.0) and access to justice (“other” 7.8). Ratings for speeding up procedures range from 5.0 (notaries) to 7.3 (“other”). Cost reduction ratings vary from 5.0 (judges) to 6.7 (lawyers), except notaries, who rate it low (2.0).
  Satisfaction differs by group: notaries rate automated case allocation and online payments very high (10.0), lawyers appreciate e-filing (7.6) and online payments (7.4), judges show moderate satisfaction but low for online dispute resolution (2.0), and administrative staff rate dispute resolution and online payments very low (1.5). Thus, electronic case management and e-filing are generally well-rated, while online dispute resolution needs improvement across all groups.
- **Predictive justice familiarity and acceptance (PJf&a):** Administrative staff have the highest familiarity with predictive justice (6.4), followed by the “other” category (4.1). Lawyers (3.2), notaries (3.0), and judges (2.2) report lower familiarity. Administrative staff are most positive about predictive justice (7.4), with the “other” category and lawyers showing moderate support (5.7 and 5.2), and notaries least favorable, but slightly positive (4.8).
  Administrative staff rate impacts highest (7.2–7.8), especially for administrative efficiency (6.4–7.8 across groups). Faster case resolution scores moderately (5.4–7.4). Notaries are skeptical, particularly about access to justice (4.6–7.2). Judges are most resistant (7.0 by lawyers, 6.3 by judges), court staff moderate (5.0–6.0), lawyers fairly resistant (4.4–5.9), and ICT personnel least resistant (3.2–5.1).

#### 3.2.3 Czech Republic

**Figure 6:**
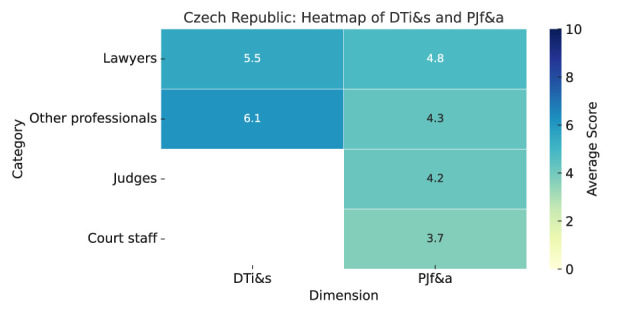
Czech Republic—Heatmap.

As summarized in Heatmap (Figure 6), the results are as follows:

- **Digital tools impact and satisfaction (DTi&s):** Judges and court staff mostly did not respond regarding digital tools' impact on justice. Among others, evaluations were generally negative, with scores below 5. The “other professionals” group strongly agreed that digital tools had no impact (7.6). Lawyers were most critical, with the highest mean score of 3.0 for “improved access to justice.”
  Both judges and court staff only expressed satisfaction with e-filing (mean 7.0). Lawyers rated electronic case management highly (6.5), while “other professionals” were dissatisfied (3.7). Conversely, “other professionals” highly rated automated case allocation, with lawyers not responding on this item.
- **Predictive justice familiarity and acceptance (PJf&a):** All professional groups show very low familiarity with predictive justice, with lawyers scoring highest at 2.5. Overall, responses are unenthusiastic; lawyers and other professionals slightly exceed the midpoint (5), with lawyers at 5.7 and judges most skeptical (2.7).
  Opinions on its impact vary. Transparency is viewed negatively, with court staff rating it highest but still low (4.0). Access to justice is cautiously optimistic, with lawyers scoring 5.0. Judges show the most confidence in faster case resolution (7.3), shared broadly. Judges are seen as most resistant (6.3), confirmed by judges themselves (7.7), and view court staff as highly resistant (7.7), more than court staff's self-rating (4.9).

#### 3.2.4 Estonia

**Figure 7:**
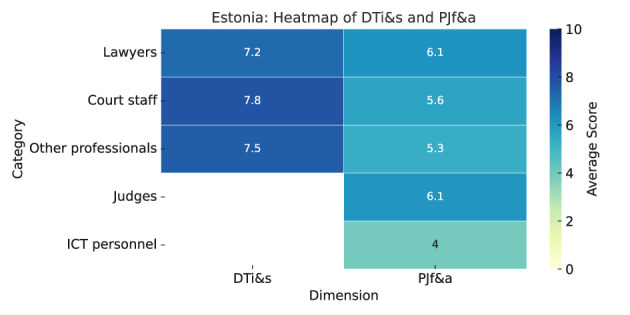
Estonia—Heatmap.

As summarized in Heatmap (Figure 7), the results are as follows:

- **Digital tools impact and satisfaction (DGi&s):** No judges responded to this section. Court staff have an extremely positive view of digital tools' impact on justice, with high scores across all benefits. The “other” category also reports relatively high averages, especially for improved access (8.0), speeding up procedures (7.0), and transparency (7.5), but lower for reduced costs (4.5). Lawyers report consistently high scores as well (access 7.0, speeding up 7.4, costs 7.4, transparency 7.2).
  Court staff show high satisfaction with digital systems (electronic case management 7.0, e-filing 9.0, online payments 10), but lower for online dispute resolution (5). The “other” group reports high satisfaction (case Management 8.0, e-filing 7.5, dispute resolution 10, online payments 7.5). Lawyers' satisfaction varies from 8.6 (online payments) to 5.7 (online dispute resolution).
- **Predictive justice familiarity and acceptance (PJf&a):** Familiarity with predictive justice is generally low in Estonia (mean 3.9). Court staff report the lowest familiarity (3.1), lawyers the highest (4.9). Judges and court staff are most receptive (6.5 and 6.6), lawyers are more cautious (5.6), and “other” professionals are most skeptical (5.3).
  All groups agree that predictive justice benefits faster case resolution, especially for court staff (8.1). It also improves administrative efficiency (lawyers 7.6, judges 6.8, court staff 7.1). Transparency improvement is doubted (scores around 4.7–5.4).
  Lawyers see judges as most resistant (7.8), while ICT personnel show the least resistance (3.1–4.8), likely due to tech familiarity.

#### 3.2.5 Italy

**Figure 8:**
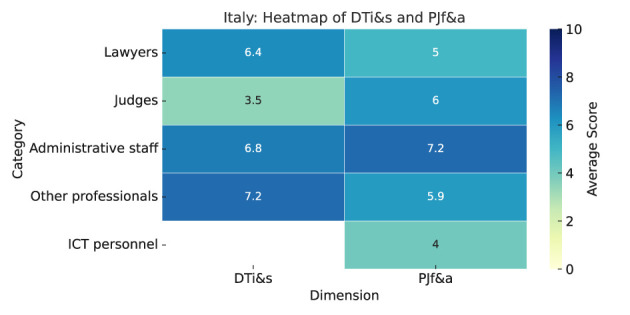
Italy—Heatmap.

As summarized in Heatmap (Figure 8), the results are as follows:

- **Digital tools impact and satisfaction (DTi&s):** The perceived impact of digital tools in the Italian justice system varies by role. Administrative staff report the highest scores, especially for improved access and transparency (both 8.0). Lawyers perceive lower benefits, particularly for reduced costs (3.7). Judges give moderate ratings but believe digital tools do not reduce costs (3.4) or have no impact (5.0). The “other” category rates impacts positively, from 7.0 (cost reduction) to 7.5 (access to justice).
  Lawyers show the highest satisfaction with online payments (7.8) and e-filing (7.6). Judges report low satisfaction with online payments (2.3) and dispute resolution (3.2). Administrative staff satisfaction varies, from 7.0 (online payments) to 4.0 (automated allocation). The “other” category consistently rates tools around 7.
- **Predictive justice familiarity and acceptance (PJf&a):** Lawyers report the lowest familiarity with predictive justice (5.2), while judges (6.5), administrative staff (6.6), and “other” professionals (6.7) report higher scores. Lawyers are most skeptical about its use in court decisions (3.9), judges are neutral (5.2), and administrative staff are favorable (6.8).
  Administrative staff rate benefits highest, especially for faster case resolution (8.2) and cost reduction (7.0). Lawyers give conservative ratings (3.8–5.1), while judges and others hold moderate views. Faster case resolution is rated highest overall.
  Judges are seen as most resistant (7.9), followed by administrative staff (7.4) and lawyers (6.8). Court staff and ICT personnel show lower resistance.

#### 3.2.6 Lithuania

**Figure 9:**
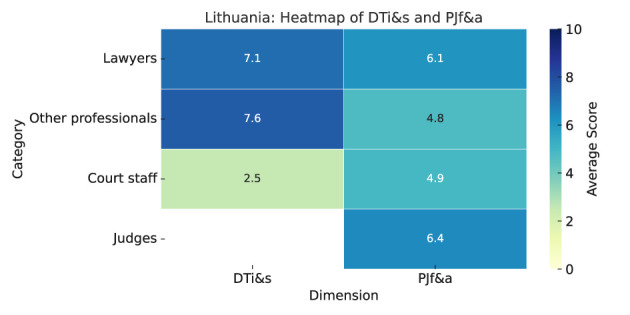
Lithuania—Heatmap.

As summarized in Heatmap (Figure 9), the results are as follows:

- **Digital tools impact and satisfaction (DTi&s):** Lawyers and other professionals rate digital tools highly for improving access to justice (7.6 and 7.0), while court staff score much lower (3.0). Both lawyers and others agree that digital tools reduce costs (7.8 and 7.0), but court staff disagree (2.5). On speeding up procedures, lawyers and others give high scores (8.1 and 8.0), while court staff strongly disagree (2.5). Lawyers strongly agree on improved transparency (7.7), while others are more cautious (6.0). Lawyers and court staff largely disagree that digital tools had no impact (2.2 and 1.5).
  Judges did not respond. The “other” category highly rates online dispute resolution and online payments (both 9.0). Lawyers are satisfied with online payments (7.8), case management (7.7), and e-filing (7.6). Lawyers are more satisfied with automated case allocation than others (7.5 vs. 7.0). Court staff report consistently low satisfaction, highest for e-filing and case allocation (3.5) and lowest for dispute resolution (1.0).
- **Predictive justice familiarity and acceptance (PJf&a):** Familiarity with predictive justice is generally good among Lithuanian legal professionals, with all mean scores above 5. Lawyers report the highest familiarity (5.9), followed by judges (5.2), court staff (5.1), and others (5.0). Judges are most receptive (6.3), lawyers are moderately positive (6.0), while court staff and others remain skeptical (around 5).
  Except for court staff's lower ratings on cost reduction (4.8) and transparency (4.2), most scores exceed 5. Judges are most satisfied with administrative efficiency (8.0), lawyers with faster case resolution (7.2), court staff also with faster resolution, and others with cost reduction.
  Judges see court staff as most resistant (6.3), while the “other” group rates resistance lowest (3.0).

#### 3.2.7 Results of the cross-country insights

**Figure 10:**
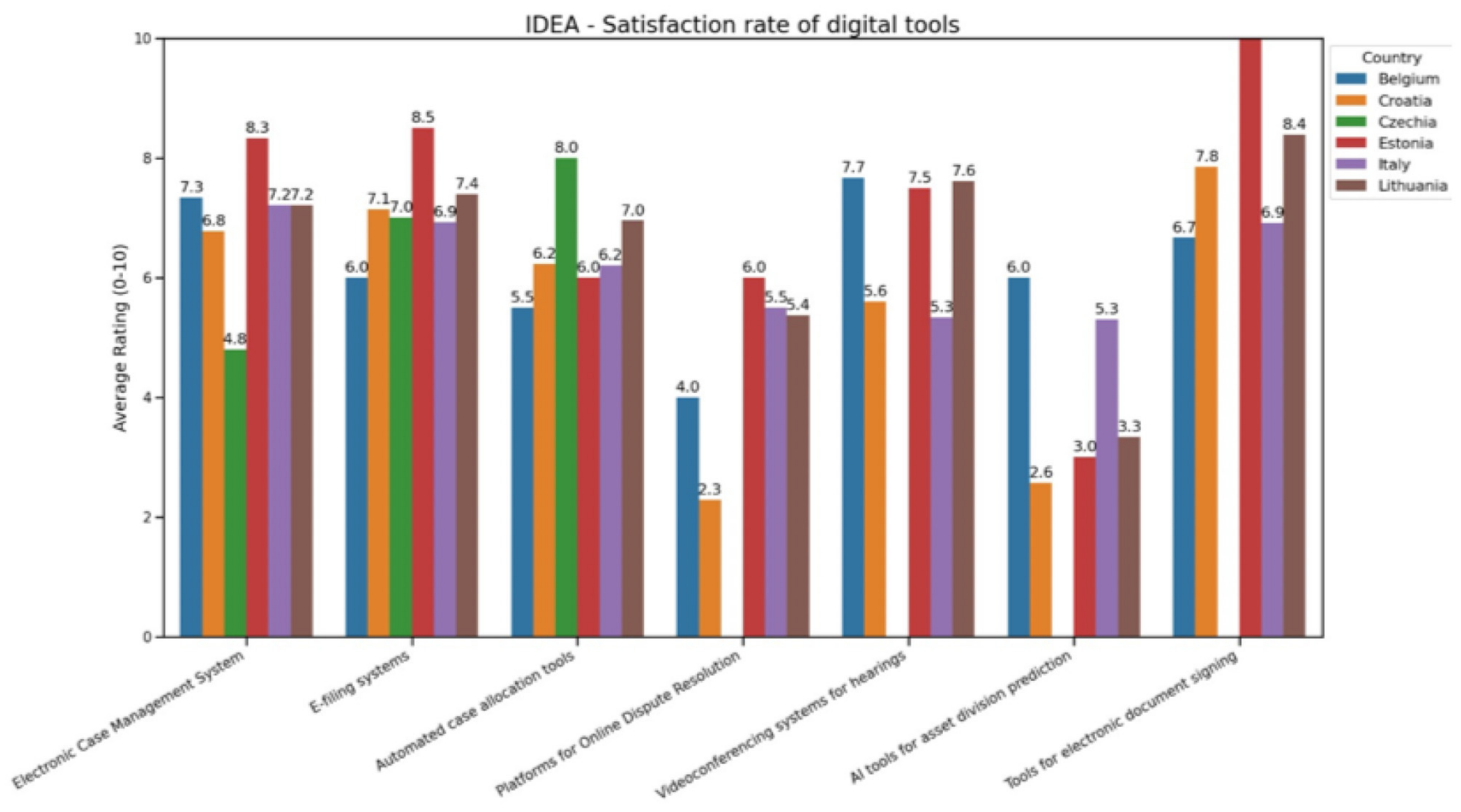
Variation in satisfaction with various digital tools.

Figure 10 highlights notable differences in how satisfied legal professionals are with various digital tools used across different countries.^9^

Electronic case management systems and electronic document signing tools generally receive high satisfaction ratings, with Estonia (8.4) and Lithuania (8.0) scoring the highest. These tools are considered well-established and smoothly incorporated into judicial procedures. On the other hand, satisfaction with more advanced technologies like predictive and online dispute resolution (ODR) platforms is much lower, especially in countries such as Belgium and Czechia, where ratings drop to 4.8 for e-filing systems and as low as 2.3 for ODR platforms.

This gap suggests difficulties in effectively embedding these newer technologies into judicial workflows, with concerns centered on their ease of use, effectiveness, and the tangible benefits they provide in case handling.

**Figure 11:**
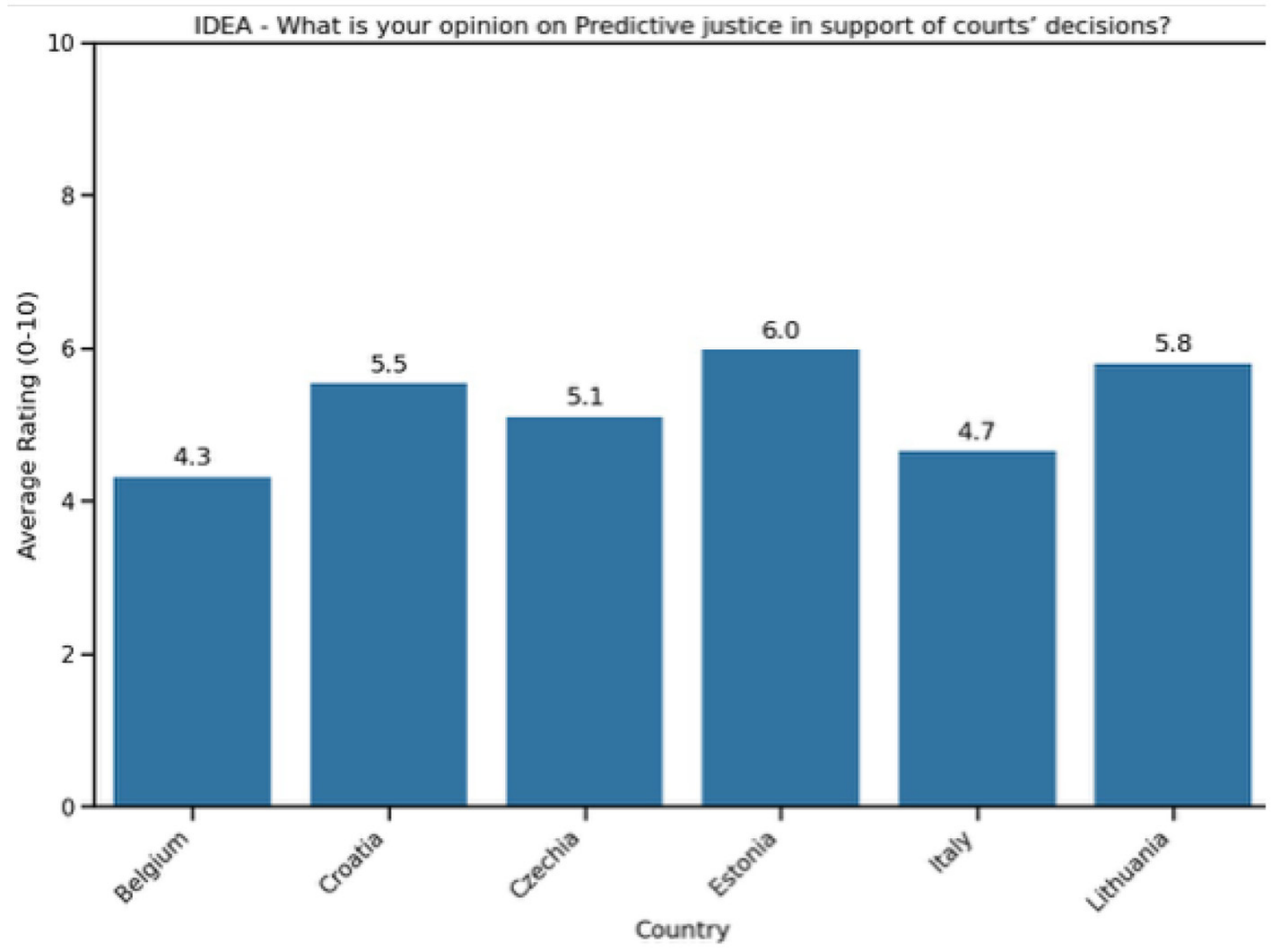
Analysis of stakeholders' attitudes toward predictive justice tools.

The analysis of stakeholder views on predictive justice tools (see Figure 11) reveals a range of opinions among the countries involved.^10^

Estonia ranks highest with an average score of 6.0, closely followed by Lithuania at 5.8, showing a generally favorable attitude toward using predictive justice to assist court decisions. Other nations, including Croatia (5.5), Czechia (5.1), and Italy (4.7), display moderate support, indicating a cautious but receptive stance. Belgium records the lowest rating at 4.3, reflecting considerable skepticism about predictive justice tools.

This variation highlights differing degrees of trust in algorithmic aids for judicial decision-making, influenced by each country's legal traditions, institutional preparedness, and concerns about fairness, transparency, and the risks associated with algorithmic bias.

### 3.3 Analysis of the cross-country insights

The cross-country review of digital tools in justice systems uncovers several important trends that reflect both advancements and ongoing challenges among the participating nations.

Estonia and Lithuania stand out as frontrunners in digital development, featuring more advanced and widely accepted digital tools that are deeply integrated into their judicial processes. These countries have shown a stronger ability to adopt new technologies, resulting in higher satisfaction levels reported by legal professionals.

Conversely, Belgium, Croatia, the Czech Republic, and Italy encounter greater difficulties. Although digital tools are present, these countries are still working to fully embed them into daily legal workflows. The lower satisfaction scores highlight persistent issues in making newer technologies work smoothly and reliably compared to more digitally advanced systems.

A notable trend is the significant resistance to predictive justice tools, especially in Belgium, where skepticism is most evident. The low ratings there reflect concerns over the transparency, fairness, and accountability of algorithm-driven decision-making in courts. This apprehension is fueled by fears that predictive tools might compromise judicial discretion or introduce bias, particularly in sensitive cases.

In contrast, Estonia and Lithuania have a more positive attitude toward predictive justice, likely due to greater trust in their digital infrastructure and successful experience implementing digital solutions across public services, including justice.

Another key insight relates to the usability and integration of these tools. Satisfaction depends not just on their availability but on how well they fit within judicial systems. While electronic case management and digital document signing tools receive high marks for user-friendliness and dependability, more sophisticated technologies like AI-based asset division prediction systems face hurdles in adoption and practical use. These tools are still perceived as immature or insufficiently tested in legal settings, affecting their credibility among legal practitioners.

The acceptance of digital tools is also heavily influenced by each country's specific legal and technological context. Countries with well-established digital infrastructures, such as Estonia, report higher satisfaction due to more robust and efficient systems.

In contrast, nations with less-developed digital frameworks struggle with implementation, as their legal professionals often deal with outdated or inconsistent technologies. This variation highlights the need to customize digital strategies according to each country's unique challenges and circumstances, acknowledging that a universal approach is unlikely to be effective.

### 3.4 Emerging needs

Based on these findings, several recommendations arise to further advance the digital transformation of justice systems within the countries involved in the IDEA Project:

1. First, building stronger trust in predictive justice tools is essential. In countries like Belgium, where skepticism is more evident, implementing additional safeguards to ensure transparency, fairness, and alignment with judicial standards would be beneficial. Clear documentation of the algorithms and regular audits could greatly enhance confidence in their fairness. Beyond transparency and calibrated explainability, it is crucial to make model advice actionable for non-experts and auditable by professionals, thereby aligning with emerging governance debates in EU AI law (Wachter et al., 2017).
2. Second, enhancing user training and support is critical. The relatively low satisfaction with emerging technologies such as AI for asset prediction and online dispute resolution platforms indicates the need for more targeted training. Legal professionals require comprehensive programs to fully grasp these tools and their benefits, which would enhance both adoption and satisfaction.
3. Additionally, “tailoring” is not only a matter of interface or language: it requires encoding the legally salient differences in national transposition, because those differences shape both the decision-tree the user sees and the meaning of any case-law-based signals the system surfaces (Aloisi and Gramano, 2019). Proper integration will make these tools more practical and valuable for legal practitioners.
4. Finally, promoting cross-country collaboration and sharing of knowledge could greatly benefit countries with less digital advancement. Estonia and Lithuania, for example, could act as role models by sharing their best practices and experiences. Such cooperation would help narrow the digital divide between countries, enabling them to overcome common obstacles and enhance overall satisfaction with digital justice technologies (Edwards and Veale, 2017).

## 4 Toward predictive justice: practical and normative challenges

### 4.1 Focus groups as qualitative enrichment

The empirical results, derived from the comparative analysis presented, are both quantitative and qualitative in nature. Further in-depth examination of the qualitative component is required to identify best practices currently implemented in the participating states, with the aim of facilitating a potential harmonized extension.

Surveys constitute a quantitative research strategy particularly well suited to rapid data collection, owing to their relatively low cost, scalability to large populations, and straightforward administration (Gürbüz, 2017). In contrast, within user-centered design, interviews represent a qualitative approach through which designers engage directly with users to elicit insights, preferences, and evaluative feedback (Blandford, 2013). Such interviews typically adopt structured or semi-structured formats that probe users' needs, behaviors, and motivations by examining specific facets of a product or service and its use. The resulting evidence informs design decisions, helping to ensure that the final outcome aligns with users' expectations and requirements (Wood, 1997). In parallel, focus groups—also a qualitative technique—facilitate an in-depth exploration of participants' opinions, perceptions, and experiences on a defined topic under the guidance of a moderator (Onwuegbuzie et al., 2009).

Thus, a comparative mixed-methods design is intentionally selected to address the need for both scope and nuance. Survey data furnish quantitative indicators of recurring patterns, bottlenecks, and cost structures that enable systematic cross-case comparison. Semi-structured interviews add depth by revealing legal, procedural, and user-experience dimensions that are not accessible through statistics alone. In turn, focus groups serve to corroborate emergent results and facilitate the co-creation of actionable solutions with stakeholders.

To this end, the consortium involved in the IDEA project undertook interviews focus groups specifically designed to address this objective.

Focus groups are structured discussions facilitated to explore participants' views on specific subjects. Within the IDEA project, stakeholders invited to participate include judges, court staff, lawyers, and ICT specialists with professional experience in labor-law courts, particularly related to the project's thematic areas such as:

- Perceptions of technological development in the judiciary;
- Level of training provided;
- Attitudes toward AI and predictive justice;
- Perception of AI-related risks and mitigation measures.

More in detail:

1. Perception of technological development in the judiciary:
  - How have recent introductions of digital tools (e-filing, case-management platforms, virtual hearings) changed the way you manage labor-law cases day-to-day?
  - In your experience, to what extent have these digital workflows improved (or, conversely, complicated) the efficiency and user experience of case management in your court?
2. Level of training provided:
  - Do you consider the ICT training you have received so far to be sufficient and relevant for your daily responsibilities?
3. Attitude toward AI and predictive justice:
  - Have you encountered or tested predictive models? If so, how accurate or useful were they in reflecting the outcomes you would expect from a human decision-maker?
  - What risks do you foresee in using predictive models in justice and in particular in labor law?
4. Perception of AI-related risks and mitigation measures:
  - From your point of view, what are the most pressing ethical, legal, or operational risks posed by integrating AI into labor-law adjudication?
  - Which safeguards - such as mandatory human oversight, transparency requirements, or regular bias audits - do you believe are most critical to mitigate those risks effectively?

The interviews and focus groups serve several important purposes.

Primarily, they enable the collection of firsthand experiences and perspectives from practitioners working within labor-law courts. Participants reflect on how the introduction of digital technologies has influenced their daily workflows and case management processes.

They assess whether the training they have received sufficiently equips them to effectively utilize these tools, and they discuss the perceived benefits and concerns regarding emerging AI-driven or predictive justice technologies.

These qualitative discussions help to contextualize and enrich the quantitative survey data by providing deeper insights. Furthermore, the dialogues help validate preliminary findings and contribute to the co-creation of practical recommendations that aim to at mitigate risks while upholding core principles, such as fairness and accountability (Kleinberg et al., 2018).

The insights gained from each session will directly guide subsequent research activities, ensuring that the IDEA project's outputs and recommendations remain grounded in the practical realities faced by judicial professionals across Europe.

Preliminary discussions consistently revealed profession-specific divides in acceptability: while court staff emphasized workflow gains, judges stressed due-process externalities and potential “automation bias” (Burrell, 2016). These findings align with broader international evidence and support a strict human-in-the-loop design for any predictive functionality (De Stefano, 2019).

#### 4.1.1 Critical discussion on data bias and limitations

In Belgium, eleven interviews were conducted with two judges, two lawyers, two court staff, two mediators, and three policymakers. One judge and one court staff member participated in a focus group. Judges were contacted via an internal connection, court staff through referrals from chief clerks, and lawyers and mediators through professional websites. A personal email invited participants for semi-structured interviews, ensuring gender balance.

In Croatia, data collection included two focus groups and one interview. Participants were selected to provide diverse, expert opinions, with some chosen from younger generations to reflect intergenerational disparities. The focus groups featured three lawyers (two ICT law and human rights experts, one with a senior judicial background and the other a younger lawyer undergoing ICT education) and one labor law expert, along with two senior judges with court administration experience. A junior judge participated in the interview. Regional differences were also considered in the selection process.

In the Czech Republic, focus groups and interviews involved diverse participants from the judicial system to provide comprehensive perspectives on technological development and AI integration in justice. The group included two judges (one from the district court and one from the Supreme Administrative Court), two court staff (a Supreme Court civil law assistant and a Constitutional Court assistant), two lawyers (one with experience in arbitration and domain name disputes, and one in labor law), and two ICT specialists working within the judicial system. The selection ensured representation across various court levels, professional roles, and areas of expertise.

In Estonia, researchers focused on labor committee judges rather than ordinary court judges, as under Estonian labor law, employees can either address the Labor Dispute Committee or sue directly in civil court for contractual violations. Ordinary courts serve as a “second instance” for Labor Dispute Committee decisions. In total, ten stakeholders were interviewed: five lawyers, one Labor Dispute Committee judge, three judges, and one notary.

In Italy, individuals from the initial surveys and new professionals were contacted, including one judge and three lawyers, one of whom was a labor law professor. The judge and professor agreed to a dialogue-based interview, while the others preferred brief written responses.

In Lithuania, data gathering involved two focus groups. The first group included six stakeholders: two judges, two attorneys-at-law, and two court staff serving as judges' assistants, providing insights from daily courtroom practice. The second group consisted of two court ICT experts, offering technical and systems-level perspectives.^11^

Considering all the above, the following biases and limitations can be identified:

1. Professional Bias: Participants were mainly selected based on their roles within the judicial system (e.g., judges, lawyers, ICT specialists), which may have led to self-selection bias. As a result, individuals more engaged with AI or interested in technological advancements in justice could be overrepresented.
2. Sample Size: With a relatively small sample size (6–11 participants per country), the ability to generalize the findings to a larger population is limited. The results may not fully capture the diversity of opinions across the judicial system.
3. Representativeness: Although efforts were made to achieve gender and regional balance, the sample may not adequately reflect all regions or professional levels, particularly lower-level staff or those less involved with technological innovations.
4. Format of Participation: The use of interviews and focus groups may have attracted individuals who are more comfortable with these methods, potentially excluding those less inclined to participate. This could lead to a biased sample of respondents.
5. Group Dynamics: In focus groups, participants may have been influenced by the opinions of others, reducing the variety of responses and limiting the diversity of perspectives.

#### 4.2 Chatbot design for labor law litigation support

Building on the best practices identified, the consortium involved in the IDEA project is developing a chatbot capable of recommending the most suitable option to parties engaged in litigation, whether that be automated negotiation, online mediation, or traditional settlement proceedings.

A chatbot is considered an optimal solution because it operates through a software application or web interface designed to facilitate natural language conversations, either textual or spoken, simulating human-like interaction (Cirillo et al., 2024). It primarily functions by detecting keywords or phrases in users' inquiries and responding with preloaded, accurate information

AI-enhanced chatbots, however, extend beyond simple keyword recognition by interpreting the relevant factual and legal context of a user's message. This capability enables more nuanced, personalized dialogues that adapt dynamically to the interaction's context and previous exchanges. Such sophistication is achieved through training on extensive textual datasets.

In practice, the IDEA chatbot will be designed for use by potential parties in litigation cases. Plaintiffs, defendants, or third parties will be able to pose questions to the chatbot and receive responses related to their case and its potential outcomes. Notably, the chatbot will provide comparative feedback on the costs and timeframes associated with resolving disputes through negotiation or mediation, versus pursuing resolution through ordinary court proceedings. This empowers parties to make informed, active decisions about dispute resolution, rather than deferring entirely to external adjudication (Giacalone et al., 2025).

Furthermore, the IDEA chatbot aims to demonstrate its potential in reducing litigation by improving the dissemination, transparency, and awareness of information related to legal processes. By encouraging out-of-court settlements and fostering greater party involvement, it seeks to promote social change, enhance satisfaction, and reduce the incidence of appeals (Rabinovich-Einy and Katsh, 2014).

As chatbots rely heavily on large volumes of data, the IDEA chatbot will be trained on a substantial collection of court decisions. Operationally, it will follow an agentic retrieval-augmented workflow: it will be able to generate targeted search actions over trusted legal sources, retrieve the most relevant documents, and iterate until it can provide an evidence-grounded explanation to the user.

To justify the use of AI empirically, the IDEA system would be evaluated through a two-part experimental design that mirrors its architecture: (i) the chatbot's retrieval-and-explanation component (agentic RAG), and (ii) the separate predictive module that outputs comparative estimates for ADR versus ordinary proceedings.

For the first component, a benchmark set of legally realistic redundancy scenarios would be constructed and annotated ex ante with the “reference set” of applicable sources (e.g., relevant statutory provisions, procedural obligations, and representative decisions). Performance could then be reported in terms of retrieval adequacy and explanation traceability, asking whether the chatbot (a) retrieves at least one core relevant source and (b) links its guidance to that source in an intelligible way. A simple illustration could be an accuracy-style indicator: if the system answers 10 benchmark queries and, in 8 of them, it retrieves and cites at least one key authority from the reference set, the retrieval adequacy rate is 8/10 = 80%. For the second component, evaluation could focus on prediction error for time and cost estimates by comparing outputs against observed values (where available) or expert-validated reference ranges. For example, if the model estimates a duration of 90 days and the reference duration is 100 days, the absolute error is 10 days; if it estimates €2,000 and the reference cost is €2,200, the absolute error is €200. Aggregating these differences across cases yields an average error (e.g., an average deviation of about 12 days over 100 test cases), which provides a transparent, audit-friendly measure of performance. Reporting these results alongside the sources cited and the reasoning provided ensures that the system's benefits can be assessed without treating outputs as authoritative, and remains consistent with the project's emphasis on provenance, interpretability, and governance safeguards.

Any model trained on case law inevitably learns the jurisdiction that produced that case law. Even where the EU framework is shared, the categories courts rely on, the procedural checkpoints that matter, and the remedial baselines that shape outcomes vary across MSs. That makes a single, undifferentiated “predictive layer” hard to justify in legal terms. The design implication for IDEA is a hybrid approach: jurisdiction-specific retrieval and training data on the one hand, and a transparent rule layer on the other, so that thresholds, deadlines, and information/notification duties reflect the applicable national transposition rather than statistical regularities alone.

This approach will enable the chatbot to tailor its responses in accordance with prevailing legal trends and case law reasoning. Importantly, training data will consist of anonymized or pseudonymized judgments to ensure privacy and compliance with data protection standards (Constant et al., 2024).

A pilot version of the chatbot will be tested in three court of the six EU MSs to enable user evaluation.^12^

In redundancy matters, the prototype would not forecast the behavior of individual judges or optimize forum selection; instead, it will constrain itself to doctrine and procedure aware guidance (e.g., thresholds, deadlines, information duties), thereby avoiding practices criticized in jurisdictions that curtail judicial analytics (Cortés, 2011). The interaction style privileges explainable prompts (including counterfactual suggestions) over opaque scores, and logs rationale for subsequent professional review.

#### 4.3 The IDEA chatbot as a regulatory tool in redundancy dismissal cases

This section advances a normative and practical claim: a legal chatbot, such as the IDEA prototype, can function as a genuine regulatory tool within European labor law. It does so by structuring information, sequencing procedural choices, and embedding safeguards that collectively guide parties toward compliance in redundancy dismissal contexts. Rather than adjudicating rights or substituting legal counsel, the system exercises a regulatory influence through anticipatory guidance, clarifying when consultation obligations arise, which facts are relevant, and what documentary evidence is required, while also providing ex post orientation by signaling remedial avenues and evidentiary burdens. In this sense, the chatbot enacts a form of algorithmic regulation, one in which computational logic translates legal norms into interactive pathways without usurping human decision-making (Katz et al., 2017).

The system operationalises the logic of Directive 98/59/EC and its interpretative case law concerning the notion of “establishment” and the timing of procedural duties. By posing precise questions to employers about workforce distribution, restructuring calendars, and selection criteria, it renders visible the legal implications of each course of action. When redundancies are planned across several local units, the chatbot calculates whether the statutory threshold of twenty employees is reached within each establishment and explains that consultation and notification duties apply even when company-wide numbers are higher but local counts are not. In this way, the application performs preventive clarification, thereby avoiding misclassifications that have historically led to litigation. When management transitions from merely contemplating redundancies to formally proposing them, the chatbot highlights that consultation must occur before definitive decisions are taken, thus creating a real-time compliance checkpoint that discourages procedural shortcuts.

For workers and their representatives, the tool diminishes informational asymmetry by explaining the data employers must disclose, the reasons and numbers involved, and the selection criteria used. It also maps realistic timelines for negotiation, mediation, or judicial filing, integrating recent EU digitalization instruments that facilitate cross-border communication. Furthermore, it produces counterfactual prompts—suggesting, for instance, that if specific evidence were introduced or mitigation offered, the worker's legal position would strengthen—thereby turning abstract rights into concrete strategic options. Such forms of explainability are increasingly recognized in the literature as enhancing contestability and the quality of legal decision-making (Danziger et al., 2011).

The chatbot's regulatory value also depends on its governance design.^13^

Because transposition choices change what counts as compliance—and what follows from non-compliance—the same interface can end up steering users differently across Member States unless those differences are made explicit. The governance layer should therefore include a maintained transposition map (at least for the parameters the chatbot operationalises), jurisdiction-specific versioning and change-logs, and outputs that clearly state which national implementation the guidance is based on. Where a user's scenario plausibly implicates more than one legal order (e.g., multi-site restructurings or cross-border establishments), the tool should not “smooth” the divergence: it should flag the fork, default to cautious guidance, and indicate the points at which professional advice or judicial interpretation becomes determinative.

Ultimately, the IDEA model embodies a compliance-by-design philosophy: it aims to minimize legal errors, reduce unnecessary disputes, and encourage proportionate alternatives to litigation, all while preserving judicial independence and the guarantees of a fair hearing (Dagnino and Armaroli, 2019).

## 5 Conclusions and future works

This paper offers a foundational empirical and comparative analysis of AI integration in EU labor law. Our analysis, framed within the IDEA project and based on comparative empirical data from six MSs, reveals how digital justice innovations can address critical challenges, such as improving access, enhancing efficiency, and promoting fairness, particularly in complex and sensitive cases like redundancy dismissals.

Read through a regulatory lens, the IDEA chatbot demonstrates how deliberate information design and explainable guidance can direct parties toward lawful redundancy procedures, thereby alleviating judicial workload while safeguarding procedural justice and due-process values. This approach reflects a broader movement in European digital justice toward embedding normative reasoning within technical architectures rather than delegating it to them (Bibal et al., 2021).

While the level of digital maturity and receptiveness to AI-based predictive justice varies significantly across jurisdictions, with Estonia and Lithuania emerging as leaders in digital adoption, other countries such as Belgium, Croatia, the Czech Republic and Italy demonstrate considerable resistance largely tied mainly to concerns over judicial independence, algorithmic transparency, and ethical implications. Importantly, the trust in digital tools and openness to predictive justice are heavily influenced by each country's specific legal and technological context, which shapes the degree of acceptance and successful integration of these innovations into judicial processes.

The data point to marked national disparities in both digital readiness and willingness to rely on predictive-oriented instruments. Estonia and Lithuania stand out for overall digital advancement (8.4/10 and 8.0/10) and also register comparatively higher acceptance of predictive justice (6.0/10 and 5.8/10). By contrast, Belgium (4.3/10), Croatia (5.5/10), the Czech Republic (5.1/10), and Italy (4.7/10) display weaker support: although digital solutions exist, their partial uptake in everyday legal practice coincides with more guarded attitudes toward predictive models. Routine technologies — such as electronic case-management and e-signature systems — attract consistently strong satisfaction scores in the two frontrunner jurisdictions, indicating that these instruments are mature and well embedded in judicial workflows. More sophisticated applications, including predictive systems and online dispute resolution (ODR), receive far lower evaluations, particularly in Belgium and the Czech Republic, where assessments fall to 4.8 for e-filing and to as little as 2.3 for ODR platforms.

The implementation of tailored digital tools, such as the development of a legal chatbot to assist workers in navigating dispute resolution pathways—proposed by the IDEA Project, underscores the tangible benefits of making use of AI to facilitate user-friendly and timely legal guidance. This technology holds promise not only to alleviate the workload of courts by encouraging amicable settlements and alternative dispute resolution mechanisms, but also to democratize access to justice by overcoming linguistic, cultural, and procedural barriers faced by vulnerable workers, especially in cross-border contexts.

Looking ahead, future research and development will prioritize the pilot testing of the chatbot in selected MSs, focusing on its practical usability, effectiveness in real-world settings, and impact on procedural outcomes. A crucial area of future work involves expanding the chatbot's applicability beyond labor law to encompass other litigation fields, including civil, family, and commercial disputes, where similar needs for improved access and procedural guidance exist. Such an extension would require careful customization to address the unique procedural and substantive legal characteristics of each domain, as well as the development of AI models calibrated to their specific contexts.

Additionally, advancing the ethical and transparent use of AI within judicial systems remains paramount. Future efforts will concentrate on reflecting on robust governance frameworks, including clear algorithmic accountability, data privacy protections, and ongoing stakeholder engagement, to ensure that digital justice tools support rather than undermine core legal principles (Medvedeva, 2024). Cross-national collaboration and the sharing of best practices will be vital to bridge the digital divide across European courts, enabling less digitally mature systems to learn from frontrunners and fostering greater harmonization of digital justice standards across the EU.

In summary, this study lays a critical foundation for the digital transformation of justice in the European Union, demonstrating that AI and digital tools can significantly contribute to making labor law litigation more accessible, efficient, and fair. By building on these insights and continuing to expand the scope and sophistication of digital solutions, future work promises to further break barriers and bridge gaps in justice delivery across a broad spectrum of legal areas.

## Data Availability

The datasets presented in this study can be found in online repositories. The names of the repository/repositories and accession number(s) can be found below: https://zenodo.org/records/17552958.

## Footnotes

1 Grant Agreement No. 101160528, 2024.

2
https://www.coe.int/en/web/cepej/special-file

3
https://curia.europa.eu/jcms/upload/docs/application/pdf/2015-04/cp150047en.pdf

4
https://employment-social-affairs.ec.europa.eu/policies-and-activities/rights-work/labour-law/employee-involvement/collective-redundancies_en

5
http://data.europa.eu/eli/dir/2001/23/2015-10-09

6
http://data.europa.eu/eli/dir/1998/59/oj

7 The heatmaps below visualize the distribution of scores for Digital Tools Impact and Satisfaction (DTi&s) and Predictive Justice Familiarity and Acceptance (PJfa) across various legal professions in the six Member States considered. Each profession is assigned an average score based on its experience and acceptance of digital tools and predictive justice systems. The heatmap operate by using color gradients to represent data values, with darker or more intense colors signifying higher scores, while lighter shades indicate lower scores.

8 IDEA Consortium, Deliverable D2.2 – International Synthesis Report JUST-2023-JACC-JUSTICE (Grant Agreement NO. 101160528, 2024).

9 The categories include electronic case management systems, e-filing systems, automated case allocation tools, platforms for online dispute resolution, videoconferencing systems for hearings, AI tools for asset division prediction, tools for electronic document signing. The bars represent average satisfaction scores (on a scale of 1 to 10) for each country, providing a comparative view of how different countries rate their digital tools in the judicial system.

10 The ratings, ranging from 0 to 10, reflect how participants in each country perceive the role of predictive justice in judicial decision-making.

11 IDEA Consortium, Deliverable D2.3 – Final Report and Guidelines JUST-2023-JACC-JUSTICE (Grant Agreement NO. 101160528, 2024).

12 The IDEA chatbot is currently under development, with progress aligned with the specific phases of the related project. At this stage, preliminary pilot results, interaction examples, and a risk assessment are not yet available.

13 The pilot testing, which will take place in three different MSs, will also focus on the national divergences in the transposition of EU law, which may impact the performance of the chatbot and its algorithmic regulatory functions.
